# Electrically Driven Plasmons in Metal–Insulator–Semiconductor Tunnel Junctions: The Role of Silicon Amorphization

**DOI:** 10.1021/acs.nanolett.2c04863

**Published:** 2023-03-01

**Authors:** Omer Erez-Cohen, Olga Brontvein, Israel Bar-Joseph

**Affiliations:** † Department of Condensed Matter Physics, Weizmann Institute of Science, Rehovot 7610001, Israel; ‡ Department of Chemical Research Support, Weizmann Institute of Science, Rehovot 7610001, Israel

**Keywords:** electrically driven plasmons, inelastic tunneling, tunnel junctions, amorphous silicon, two-electron tunneling

## Abstract

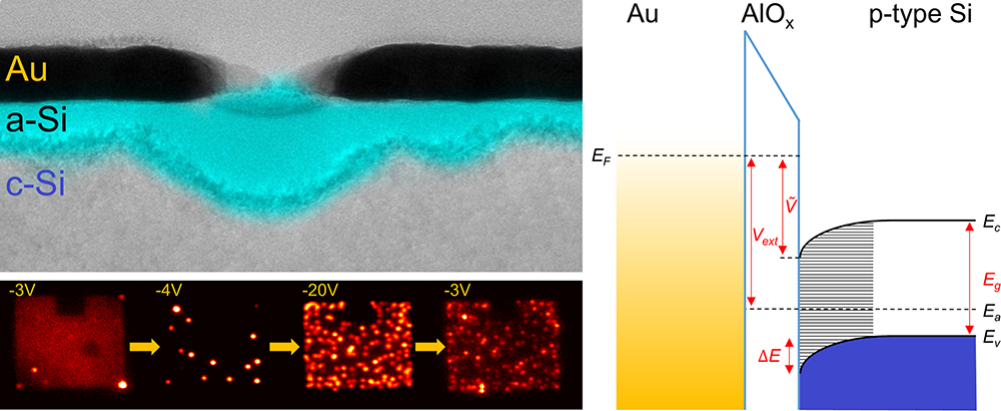

We investigate electrically driven plasmon (EDP) emission in metal–insulator–semiconductor tunnel junctions. We find that amorphization of the silicon crystal at a narrow region near the junction due to the applied voltage plays a critical role in determining the nature of the emission. Furthermore, we suggest that the change in the properties of the insulating layer above a threshold voltage determines the EDP spatial properties, from being spatially uniform when the device is subjected to low voltages, to a spotty pattern peaking at high voltages. We emphasize the role of the high-energy emission as an unambiguous tool for distinguishing between EDP and radiative recombination of electrons and holes in the semiconductor.

Electrically driven plasmons (EDP) in metal–insulator–metal (MIM) structures^1,2^ have been studied extensively in both theoretical and experimental frameworks.^3−15^ Inelastic tunneling of electrons in these structures subjected to a voltage *V* may excite a surface plasmon with an energy *ℏω* ≤ *eV*, which can scatter at the surface and yield a far field photon. The appeal of integrating plasmonic devices in ultrafast communication and their potential application as effective on-chip emitters with electrically tunable spectra have triggered exploration of methods for realization of EDP devices in metal–insulator–semiconductor (MIS) structures.^16−19^ Plasmon generation in such structures occurs as a result of inelastic tunneling of electrons from the metal layer into unoccupied states in the semiconductor, which are in either the conduction or valence bands.

The presence of an energy gap, *E*_*g*_, the type of doping, *p* or *n*, and the band bending in the semiconductor near the junction, Δ*E*, should influence the emitted spectrum in MIS devices. If no holes are present near the junction, electrons may tunnel into the conduction band only. In this case, both the initial and final states are a continuum, and at a zero-temperature approximation the EDP spectrum, *S*(ω), should resemble that of an MIM device,^20^ such that *S*(ω) ∼ ρ(ω)(*eṼ* – *ℏω*). Here ρ(ω) is the device plasmonic scattering spectrum and *Ṽ* is the energy drop across the junction, which in general may differ from the applied voltage, *V*_*ext*_, because of band bending. It can be readily seen that *S*(ω) should drop with energy and exhibit a cutoff at *ℏω*_0_ = *eṼ* similarly to EDP in MIM devices. If holes are present, inelastic tunneling into an unoccupied valence band state may occur, and at the limit of low hole density, the emission spectrum can be approximated by *S*(ω) ∼ ρ(ω)*f*_*FD*_(*ℏω*, *eṼ*), where *f*_*FD*_ is the Fermi–Dirac distribution function of the electrons in the metal.^21^ The linear drop with energy *eṼ* – *ℏω*, which characterizes the tunneling to the conduction band, is replaced by the step-function like behavior of the Fermi–Dirac distribution.

However, a few observations cast doubts upon the understanding of EDP in MIS structures:

- Most experimental demonstrations show a highly nonuniform emission pattern,^16−18^ consisting of a finite number of bright spots.
- In some cases, a very high voltage, which may exceed 10 V, is applied on the tunnel junction, yielding photons in the visible–near-infrared range.^22,23^ Clearly, under these conditions, the simple predicted relation between the emitted photon energy and voltage is not found.
- Finally, while silicon has an indirect band gap, it is known that under certain conditions one may obtain electroluminescence (EL) in Schottky barrier diodes.^24^ A conclusive proof that the emission is due to EDP is therefore needed.

In this work we investigate EDP in an MIS device consisting of Au–AlO_*x*_–p-Si layers and find that amorphization of the silicon crystal, which occurs at a narrow region near the junction due to the applied voltage, plays a critical role in determining the nature of the emission. At low voltages, the defect states due to dangling bonds in the amorphous layer are filled by electrons, bending the bands downward near the junction. Inelastic tunneling can only occur into the conduction band, resulting in EDP emission which is nearly uniform over the entire mesa, and exhibits a linear drop with energy. We show that, beyond a certain threshold voltage, the amorphous layer becomes insulating, and most of the applied voltage drops on this layer. Under these conditions, inelastic tunneling can occur only into a narrow depletion layer near the junction, with a large hole density in the valence band. Since electrical conduction from this depletion layer into the contacts should go through the insulating amorphous layer, it can only take place in a small number of breakdown regions, giving rise to the spotty emission pattern. Finally, we examine the high-energy tail of the emission spectrum, at *ℏω* > *eV*, and find substantial emission at these energies in both cases. We show that this emission is due to two-electron tunneling processes, thus unambiguously proving that the emission is due to EDP rather than electron–hole recombination in the silicon.

To fabricate the devices, we start with a boron-doped *p*-type silicon (*N*_*a*_ = 10^15^ cm^–3^) substrate with 100 nm thermal oxide (silica) deposited on top. We open a 40 × 40 μm^2^ square window in the silica by using optical lithography and chemically etching with HF acid, exposing the bare Si substrate. The wafer is then placed in an atomic layer deposition (ALD) system, where 4 nm of alumina (AlO_*x*_) are deposited. We then use e-beam lithography to write a 35 × 35 μm^2^ square containing a periodic array of holes. The device for which the results in the manuscript are given has 120 nm holes and a center-to-center distance of 220 nm. We form the top electrode by evaporating 30 nm of Au (and a 1 nm Ti adhesive layer) on top of the alumina. A large aluminum pad is evaporated on another etched window in the silica, allowing ohmic contacts to the doped Si wafer. A schematic diagram is provided in the Supporting Information (SI Figure S1).

Throughout this work, the bias voltage is applied on the metal electrode, keeping the silicon grounded, such that electrons are fed to the device through a biased lead and tunnel through the thin alumina into the silicon. Figure 1a shows a SEM image of a typical device, with a close-up view of the top metal electrode in the inset. Imaging and spectral measurements are conducted using an oil immersed 60× objective, coupled into a Shamrock SR-500i spectrometer and imaged with an Andor iXon Ultra 897 CCD. The spectra shown in the paper are normalized by the spectral response of the CCD and spectrometer. The current–voltage (*I*–*V*) characteristic exhibits a clear diode-like behavior, reflecting the asymmetry of the structure (Figure 1c). We find that the *I*–*V* exhibits a sudden irreversible change above a certain voltage threshold, typically ∼−3 V, manifested in an increase of the current through the devices by a factor of 3–10. We therefore took special care to study the devices both before this breakdown threshold and beyond it.

**Figure 1:**
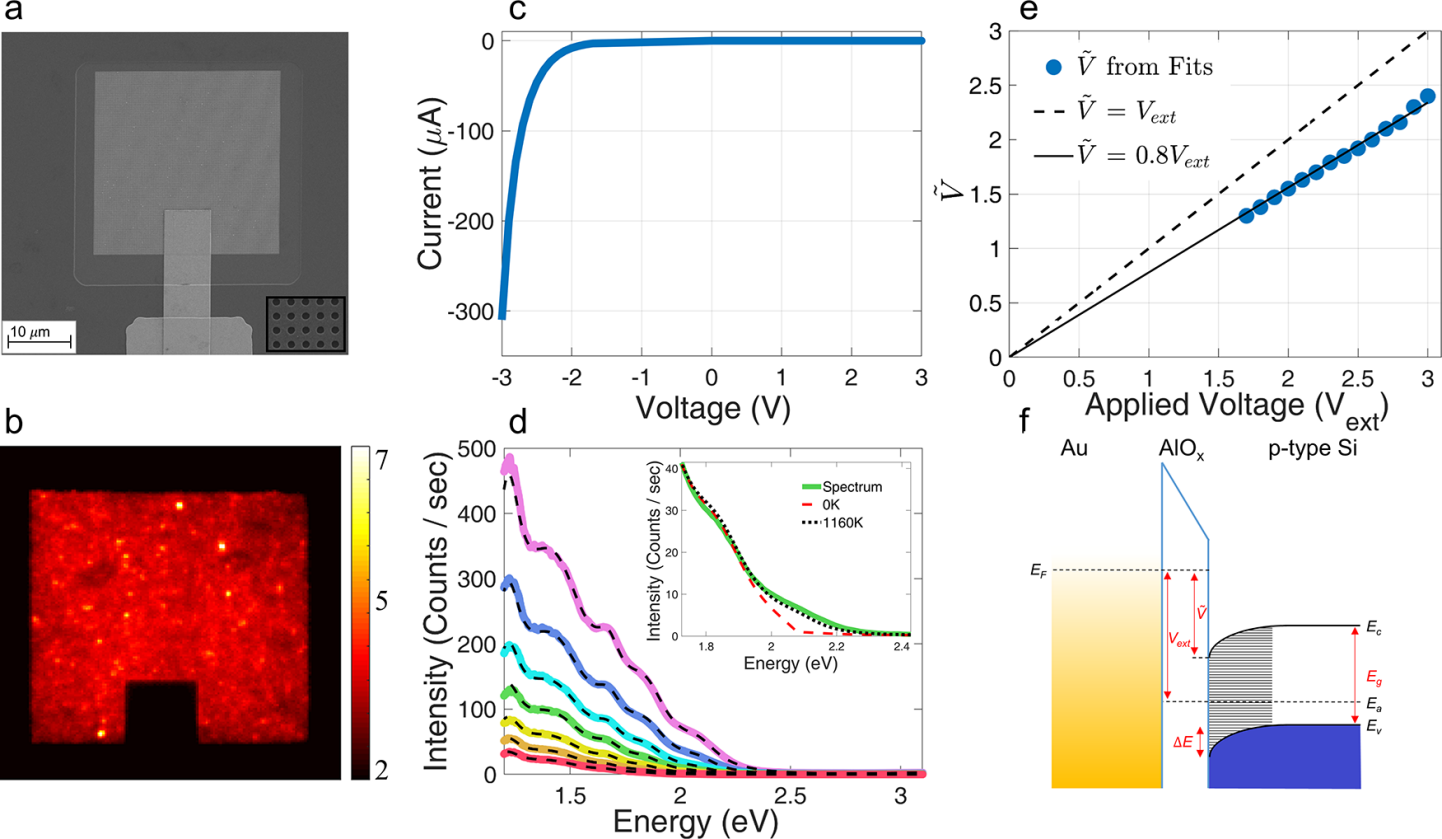
(a) SEM image of a typical device. The inset shows a close-up of the patterned array of holes. (b) Image of the emission from the device, integrated over the spectral range 400 nm–1100 nm, at an applied voltage of −3.0 V. The scale bar is in counts/s. (c) A plot of the current vs voltage of the device. (d) Measured spectra (solid) of the device in the range of −2.4 V to −3.0 V in steps of 0.1 V normalized by the spectrometer and CCD efficiency, along with theoretical fits (dashed lines). Spectra for applied voltages below −2.4 V are omitted for a clearer visualization and included in the Supporting Information (Figure S2). The inset shows the spectrum at −2.7 V and fits with finite and zero-temperature. (e) The fitted voltage drop on the junction, *Ṽ* (blue dots), as a function of the applied voltage. The dashed line depicts a case where *Ṽ* = *V*_*ext*_. (f) Schematic band diagram of the device under bias.

Figure 1b,d shows the emission image and spectra, respectively, prior to this threshold. The image clearly shows that the whole mesa emits relatively uniformly, with the intensity of the brighter spots lying within a factor of 2 of the rest of the sample. It is seen that the EDP spectra, normalized by the spectrometer and camera response, can be well approximated by a linearly decreasing function of energy, with small modulations due to the plasmonic scattering spectrum, ρ(ω). The dashed lines in Figure 1d are fits to the measured spectra, using *S*(ω) ∼ *αρ*(ω)(*eṼ* – *ℏω*), where α and *Ṽ* are fit parameters, and ρ(ω) is a multi-Gaussian function, which is the same for all spectra (see Figure S4 in the Supporting Information). We find that α is approximately linearly proportional to the measured current through the device, *I*. The electron temperature affects only the spectral region near *ℏω* ≈ *eṼ*. This is demonstrated in the inset of Figure 1d, where we compare a *T* = 0 fit to that at a finite temperature (the expression for *S*(ω) at a finite temperature can be found in the SI). The resulting electron temperature in the gold electrode is rather high, *T*_*e*_ ∼ 1000 K, in line with the findings of other measurements of *T*_*e*_ in tunnel junctions.^11,21^

The fitted value of *Ṽ* yields the voltage drop across the junction and allows us to obtain an insight into the silicon band bending near the junction. The blue dots in Figure 1e show the dependence of *Ṽ* on the applied voltage *V*_*ext*_. For comparison, we show in a black dashed line the case where the voltage across the junction is equal to the applied voltage, e.g., *Ṽ* = *V*_*ext*_, and it is clearly seen that *Ṽ* < *V*_*ext*_. A linear fit (solid black line) to the extracted values shows that *Ṽ* ≈ 0.8 × *V*_*ext*_. One can, hence, construct the band bending in the silicon region, which is consistent with these observations (Figure 1f): Far away from the junction, the chemical potential is above the valence band, at an energy which is *eV*_*ext*_ below the Fermi level of the metal. Near the junction, the bands bend downward by Δ*E* = (*E*_*g*_ – *E*_*a*_) + *Ṽ* – *V*_*ext*_ = (*E*_*g*_ – *E*_*a*_) - 0.2*V*_*ext*_, where *E*_*a*_ ≈ 250 meV is the silicon Fermi level for a doping *N*_*a*_ = 10^15^ cm^–3^ at 300 K. At zero bias, Δ*E* = (*E*_*g*_ – *E*_*a*_) and the chemical potential of the Au electrode aligns with the energy of the silicon conduction band, *E*_*c*_, at the junction, such that *Ṽ* = 0. Clearly, a high density of negative charges has to accumulate at the silicon region near the junction to yield such a large bending. As the voltage increases, the conduction band energy at the junction moves to lower energies. However, as the accumulated charge is depleted, the band bending decreases, yielding *Ṽ* < *V*_*ext*_.

To identify the source of the accumulated charges, we conducted studies of the atomic structure of a cross-section of the device near the junction. We used a Helios 600 focused ion beam (FIB) to produce lamellae of our device and a Talos F200X scanning transmission electron microscope (S/TEM) operated at 200 kV for bright-field STEM and TEM imaging. To corroborate the compositional integrity of our tunnel junctions, we use a large solid angle X-ray detector for energy-dispersive X-ray spectroscopy (EDS) analysis.

In Figure 2 we compare a pristine device, to which no voltage was applied (Figure 2a), with a device that was subjected to a voltage of −20 V (Figure 2b). It can be seen that in the device that was subjected to a high voltage the silicon crystal consists of two regions, separated by a dark border, which is significantly closer to the surface below the gold covered areas compared to the holes. This separation into two regions was also found in lamellae of devices subjected to lower voltages, *V*_*ext*_ = −2 V and −3 V. On the other hand, we do not find it in the lamella of the control device, which had no bias applied to it, implying that this feature is not a product of the device fabrication or FIB milling but rather due to the voltage applied to the device.

**Figure 2:**
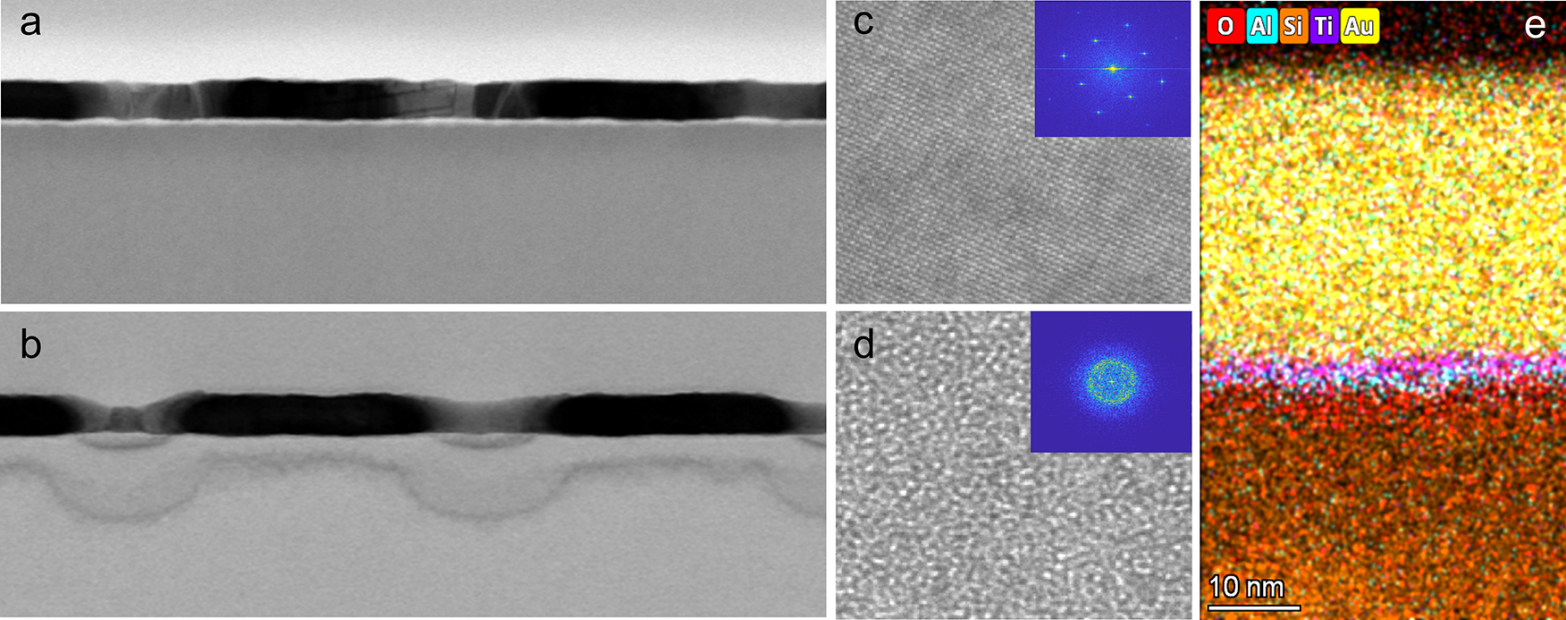
(a) Bright field (BF) STEM images of cross sections of a device taken before (a) and after (b) voltage was applied. The formation of an amorphous layer in the silicon region near the junction in (b) is evident. (c, d) BF TEM close-ups of the crystalline and amorphous regions, respectively. The insets show the corresponding Fourier transforms, indicating directional periodicity in (c) and a lack of it in (d). (e) Energy dispersive X-ray spectroscopy (EDS) elemental map combination of the tunnel junction after voltage is applied.

Figure 2c,d provides a TEM close-up view of the silicon crystal below and above the border, respectively. The periodic structure of the silicon atoms, which are indicated by white spots, is clearly seen in Figure 2c. However, this periodicity is less profound in the region close to the junction (Figure 2d), and it is apparent that it exhibits some disorder. To resolve the long-range order of the silicon atoms in the two regions we perform a Fourier transform of large areas below and above the border (insets of Figure 2c,d). We find that the region far away from the junction exhibits a series of sharp peaks arranged in a square lattice, indicating the existence of an ordinary silicon crystal (c-Si). The region closer to the junction, on the other hand, exhibits a circular ring of broad peaks, a signature of amorphous silicon (a-Si). We find that the width of the amorphous layer increases with applied voltage; when a voltage of −2 V was applied to the device, the a-Si depth is ∼5 nm under the Au electrode and ∼20–35 nm under the holes, whereas at −3 V the depths are 15–25 nm and 60–65 nm, respectively. It appears that the gold electrode serves as an efficient heat radiator, reducing the lattice temperature below the region covered with gold, giving rise to a smaller amorphization depth in comparison to regions under the holes. We measure the depth of amorphization at −20 V and conclude that, past −3 V, the amorphization remains fairly constant (Figure S12).

An elemental analysis of a device subjected to an applied voltage of −20 V (Figure 2e) shows no evidence for diffusion of Au, Si, Al, and O from their zero voltage positions, which would appear to indicate that the MIS junction remains intact. An EDS scan along the junction showing the spatial distribution of each element can be found in Section 13 of the Supporting Information.

The formation of an a-Si layer below the junction allows us to understand the origin of the large downward band bending near the junction. Since not all the atoms within the a-Si structure are 4-fold coordinated, some would have a dangling bond, which can be viewed as a defect or a localized gap state. The density of these gap states can be high, reaching a value of ∼10^19^ cm^–3^. In a steady state, electrons from the Au electrode tunnel through the barrier into the a-Si region and fill these gap states, charging them negatively and bending the bands downward by Δ*E*. With increasing voltage, the charged region is gradually depleted and Δ*E* decreases, as observed experimentally.

A close examination of the spectra shown in Figure 1d reveals that the emission is not terminated at the cutoff energy, ℏω_0_ = *Ṽ*. Instead, there is a substantial emission tail at higher energies, which extends all the way to the maximal detection energy, *ℏω* = 3.1 eV. In Figure 3 we show a close-up view of the emission spectra at high energies, for *V*_*ext*_ = −2.8, −2.9, and −3*V*, which correspond to *Ṽ* = −2.2, −2.3, and −2.4*V* (see Figure 1e). One can clearly observe a broad emission spectrum at *ℏω* > *V*_*ext*_, which gains strength as the applied voltage (and consequently the current through the device) increases. Such high-energy emission was previously observed in STM experiments^25^ and atomic junctions^26^ and was shown to be due to two-electron tunneling. When the mean time interval between two single-electron tunneling events becomes shorter than the plasmon lifetime (∼10^–14^ s), there is a contribution due to a coherent two-electron tunneling process, in which each electron contributes an energy ≤ *eṼ*. The EDP spectrum due to this process can be expressed as^27^
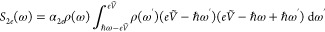
The dashed lines in Figure 3 are fits using this model, where the only free parameter is α_2*e*_. The inset in Figure 3 shows the intensity of the tails from the spectra (which is proportional to α_2*e*_) as a function of the current through the device. Indeed, we find that the tail amplitude has a quadratic dependence on the current, in accordance with a multielectron process. The presence of two-electron emission unambiguously proves that the observed emission is indeed due to EDP.

**Figure 3:**
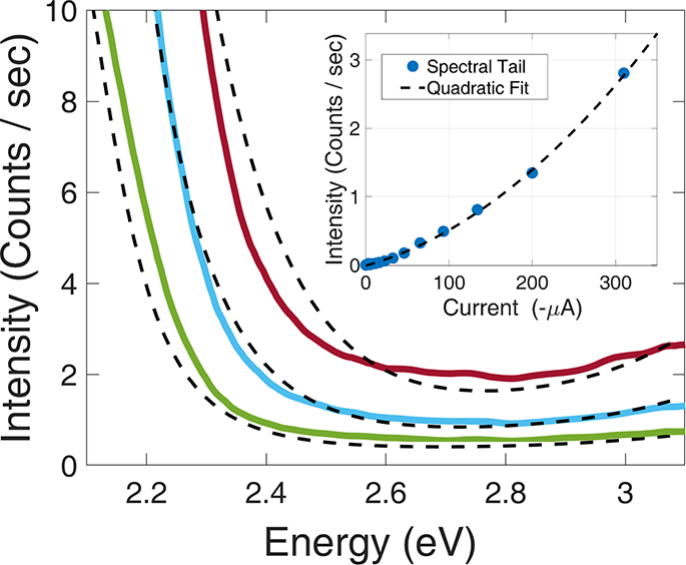
Close-up of the high-energy tails of the −2.8 V to −3.0 V spectra from Figure 1d, showing photon emission at ℏω > *Ṽ*. Dashed lines represent the fit to the data, modeling a combination of single- and two-electron tunneling. Inset: dependence of 2e^–^ emission on current with a quadratic fit.

We now turn to discuss the behavior of the device after breakdown. When a high enough voltage is applied, typically exceeding −3 V, we observe a sudden change (which we refer to as breakdown) in the electrical and optical properties of the device: The current increases significantly in an irreversible manner (Figure 4a), the uniform emission throughout the mesa disappears, and several intense bright spots appear on the device (Figure 4b). As the voltage increases further, the number and intensity of these diffraction-limited spots increase. In fact, at high enough voltage, the spots can be easily observed with a naked eye. It is important to emphasize that the number and location of the bright spots remain unchanged when the voltage is brought back down, indicating that they reflect a permanent irreversible change.

**Figure 4:**
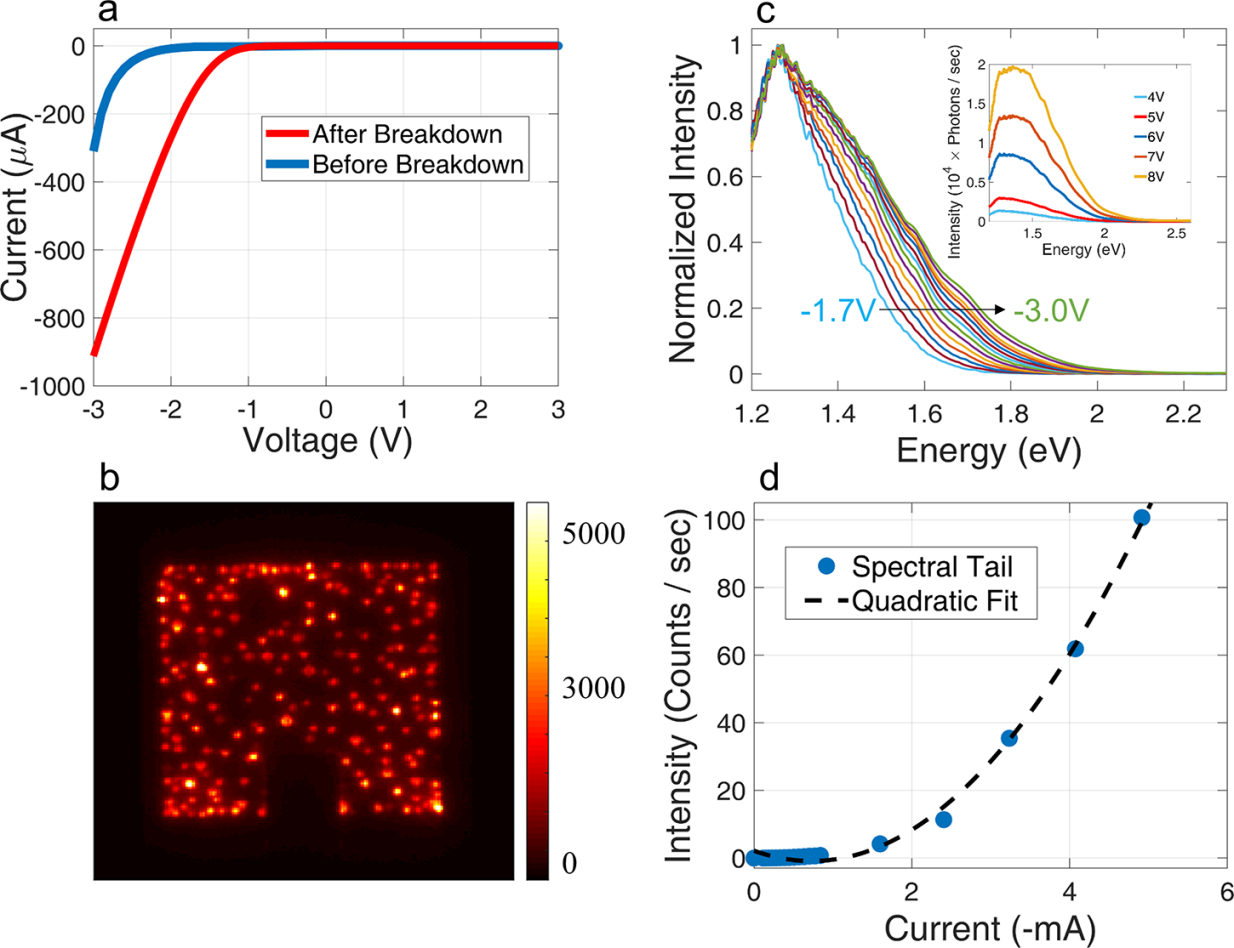
(a) Plot of the *I*–*V* curve of the device before (blue) and after (red) applying voltages past the breakdown threshold (−3.0 V). (b) Image of the photon emission of the device at high voltage (−20 V). The scale bar is in counts/s. (c) Emission spectra (normalized by each corresponding maximum) of the device after breakdown at the −1.7 V to −3.0 V voltage range. The inset depicts the high voltage spectra, in the −4 V to −8 V range, demonstrating the increasing intensity and formation of a spectral plateau with voltage. (d) The emission intensity at the highest detection energy (3.1 eV) as a function of current after breakdown. A quadratic dependence is clearly seen, indicating a two-electron origin.

In Figure 4c we present normalized emission spectra that are measured after applying a high voltage of −20 , and ramping the voltage down. TEM imaging shows that the amorphous silicon layer underneath the tunnel barrier remains nearly constant in depth (∼25 nm) after being subjected to a voltage in the range −3*V* > *V*_*ext*_ > −20*V* (Figure S11). Hence, we may consider the sample after breakdown, when ramped down to any lower voltage, as having an amorphous layer of fixed depth of 25 nm.

Let us first consider the behavior when the voltage is ramped down to low values (Figure 4c), the same as those presented in Figure 1d. It is seen that the spectra are characterized by a sharp peak, centered at ℏω ≈ 1.25 eV, and a high-energy part that broadens with voltage. We note that this peak energy coincides with the reported emission peak of a-Si,^28,29^ implying that the a-Si conduction band is pinned to the gold Fermi level at the junction. We also find that as the ramped-down voltage is large, *V*_*ext*_ < −4 V, a spectral plateau is formed at ℏω > 1.25 eV and extends to higher energies when decreasing the voltage further to larger negative values (inset of Figure 4c). This implies that a large hole density is formed near the junction at this voltage range.

With these insights from the spectra, we can construct a schematic band diagram of the device in the locations under the hot spots (Figure S9 of the SI). It is seen that the a-Si acts as an insulating layer, over which most of the applied voltage falls. The large slope of the bands in the a-Si region creates a hole accumulation layer in the valence band, to which electrons from the metal can inelastically tunnel, and gives rise to light emission. One may argue that this light emission is due to radiative recombination of the tunneling electrons with the holes in this accumulation layer, rather than due to plasmon assisted process (EDP). To examine this possibility, we study the high-energy tail of the emission. Here, again, we find a flat emission spectrum that extends all the way to the maximum detection energy of *ℏω* = 3.1 eV. Such behavior cannot be explained by radiative recombination but rather indicates that it is due to due two-electron EDP. A clear evidence that this is indeed the case comes from examining the dependence of the tail intensity on the current (Figure 4d), where a quadratic dependence is observed—a fingerprint of two-electron plasmon emission. A log scale representation of the spectra after breakdown, which provides a clear visualization of the high-energy emission, can be found in the Supporting Information (Figure S6).

To explain the appearance of the hot spots we note that, at high voltages, *V*_*ext*_ < −3 V, the electric field that drops on the 4 nm barrier, becomes comparable to the dielectric breakdown field, which is ∼1 V/nm.^30^ Hence, breakdown should occur in some local minima in the alumina layer. Indeed, atomic force microscopy (AFM) scans (Figure S15 in the SI) reveal thickness fluctuation of the alumina layer, with a standard deviation of σ = 0.36 nm. Therefore, the hot spots likely manifest the locations where dielectric breakdown of the barrier occur. When this happens, the current flows predominantly through these breakdown points, giving rise to intense emission.

In the concluding part of this paper, we wish to highlight some of the important findings of this work. We have shown that amorphization of the silicon crystal occurs already at low voltages in MIS devices with a few nm insulating barrier and can explain the emitted photon energy and applied voltage discrepancy. The changes in the properties of the alumina layer with voltage determine the EDP spatial and spectral properties: From spatially uniform and MIM-like below breakdown, to a spotty pattern peaking at the a-Si gap beyond it. This breakdown also marks the change of the inelastic tunneling final state, from the conduction band below breakdown, to the valence band beyond it. We emphasize the role of the high-energy emission at *ℏω* < *eV* as an unambiguous tool distinguishing between EDP and radiative recombination.

## Supporting Information Available

Detailed device fabrication, data from additional devices before and after breakdown, complete spectra in logarithmic scale, plasmonic spectrum, dependence of α on current, fitting at finite temperatures, hot spots evolution, band diagram after breakdown, expanded BF STEM amorphization image, amorphization measurements, EDS line profile along a junction, spectra with and without noise filters, and AFM line scan of alumina (PDF)
