# Supplementary material for: Electrically Driven Plasmons in Metal–Insulator–Semiconductor Tunnel Junctions: The Role of Silicon Amorphization
Source: Nano Lett. 2023 Mar 1;23(6):2233–8. doi: 10.1021/acs.nanolett.2c04863 (PMC10037326; doi:10.1021/acs.nanolett.2c04863)
Supplement: Supplementary file 1 — nl2c04863_si_001.pdf [file nl2c04863_si_001.pdf]

Electrically-Driven Plasmons in Metal-Insulator-Semiconductor Tunnel Junctions: The Role of Silicon Amorphization

Supporting Information

Omer Erez-Cohen[a*], Olga Brontvein[b], and Israel Bar-Joseph[a].

[a]Department of Condensed Matter Physics, Weizmann Institute of Science, Rehovot 7610001, Israel

[b]Department of Chemical Research Support, Weizmann Institute of Science, Rehovot 7610001, Israel

*Email: omer.cohen@weizmann.ac.il

Methods

We use optical lithography, electron beam lithography and atomic layer deposition to fabricate Au-AlO_x-Si tunnel junctions, where the silicon is boron-doped p-type ($N_a = 10^{15}$ cm^{-3}, verified by four-point probe measurements). We begin with a silicon substrate with a 100 nm thick thermal oxide and create 40 × 40 μm^2 windows by chemical etching with HF acid for 2-3 minutes. We write the windows using optical lithography (with a mask) and use oxygen plasma to clean photoresist residues before etching. The windows allow direct contact with the doped silicon substrate. Immediately after etching and cleaning away the photoresist (no more than several minutes, so as to prevent formation of a native oxide), we transfer the wafer to an Atomic Layer Deposition (ALD) system and deposit a 4 nm layer of alumina by running 24 cycles of an AlO_x recipe at 250° C, with TMA as a precursor. We use a Woollam M-2000 ellipsometer to verify the film thickness. A 35 × 35 μm^2 square with an array of 120 nm holes with a center-to-center distance of 220 nm is patterned on top of the alumina in each window using e-beam lithography and cold (4° C) developing for high resolution. A 1 nm adhesive layer of titanium and a 30 nm Au layer are evaporated to form the top (drain) electrodes, followed by a standard lift-off process involving hot (80°-100° C) acetone and IPA. We then sonicate in acetone for 3-4 minutes to remove leftover Au "caps". Far from the device, a large aluminum pad is evaporated directly on top of a region of etched doped silicon and annealed under argon at 460° C for 30 minutes to form an ohmic contact to the Si (this is in fact done as a first step, as the high temperatures would denature the Au). These enable using the silicon substrate as the drain electrode with minimal applied voltage loss from contact resistance. We then use the same optical lithography technique to write leads (5 nm Ti/100 nm Au) for each device, and e-beam lithography to write thick patches that bridge the leads and the

devices themselves. This step must be done with e-beam, as the photoresist developer used in optical lithography harms the integrity of the alumina barrier, leading to a shorting of the device. Since the patches must overcome the height of the thermal oxide windows, we form them by evaporating more than 100 nm of Au followed by lift-off. Finally, we contact each lead and the Al pad to a chip holder using a bonder loaded with a Au spool.

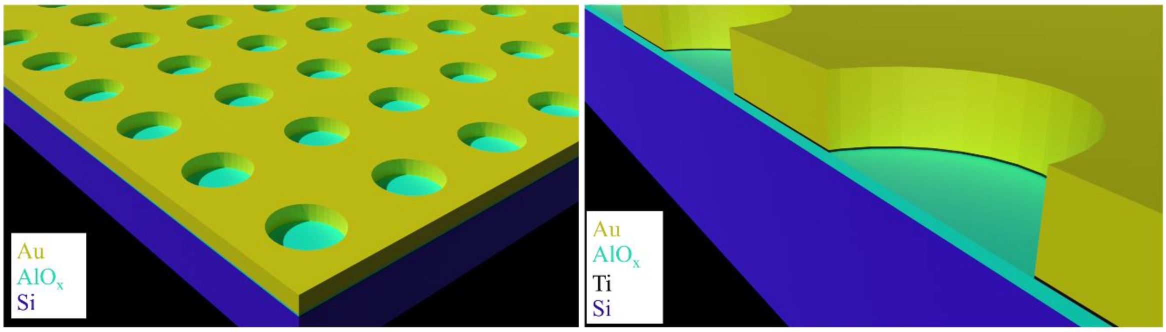

Figure S1: Schematic of the Au/AlO$_x$/Si structure (left) and close up of a cross-section of the structure showing the Ti adhesive layer as well (right)

Additional Data

1. Spectra and Images Before Breakdown

a. Expanded data for the device described in the paper

Figure S2 shows the spectra presented in Fig. 1d on a logarithmic scale for a clearer visualization. The blueshift of the cutoff energy to higher values with increasing voltage, which is the characteristic fingerprint of electrically-driven plasmonic devices, is clearly visible. The ripples observed in the emission come from the plasmonic scattering spectrum. The high-energy emission due to two-electron tunneling at $2\text{eV} < \hbar\omega$ is clearly evident. The ratio R between the intensity at high energies, $\hbar\omega \approx 3\text{eV}$, and that at low energies, $\hbar\omega \approx 1.2\text{eV}$, can be viewed as the ratio between EDP due to two and one electron tunneling. We can see that R increases as we go to higher gate voltages, manifesting the superlinear dependence of the two-electron process on the current through the device.

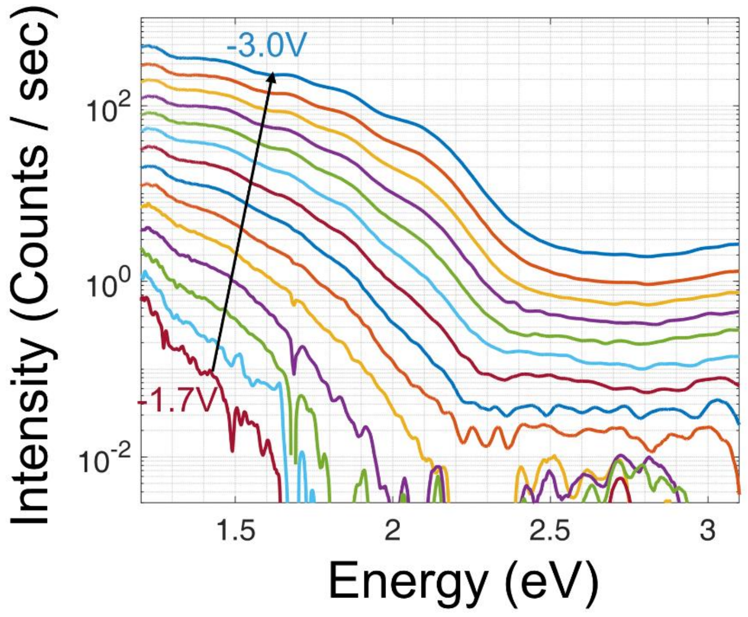

Figure S2: The emission spectrum of the device discussed in the paper for the voltage range -1.7V to -3V. The intensity is presented on a log scale.

b. Measurements of other devices

We fabricate chips that contain 20 devices each and observe the same behavior of uniform and transition to spotty emission in all the devices in all the chips that we measure, indicating that the phenomenon is robust and not a feature of chance. In Fig. S3 we present the spectral and spatial emission from two other devices and show the same behavior reported in the main device of the paper. When we measure these two device ***before*** breakdown, the emission is uniform throughout the entirety of the large device, and the emission manifests a linear decaying component $(eV - \hbar\omega)$ modulated by a plasmonic scattering spectrum $\rho(\omega)$. The device pertaining to Fig. S3 a and b has a different geometry from that of the device presented in the paper. This is manifested in the different resonant modes that modulate the emission spectra of the device.

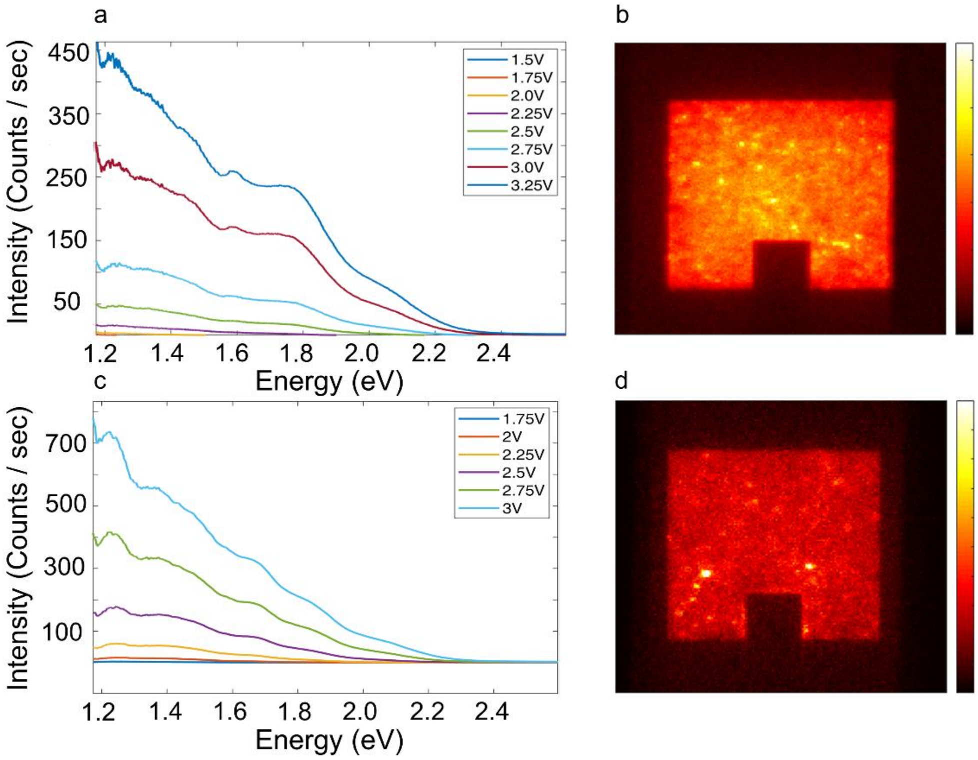

Figure S3: Spectra (a and c) and respective emission maps (b and d) of additional devices before breakdown.

2. The Fitting of the EDP Spectrum

a. The plasmonic scattering spectrum $\rho(\omega)$

The fits to the EDP spectra (dashed lines in Fig. 1d) are conducted using the same plasmonic spectrum $\rho(\omega)$ for all spectra, from -1.7V to -3V. We construct $\rho(\omega)$ as a sum of five Lorentzians, where each Lorentzian $L_j(\omega)$ is characterized by a certain amplitude A_j, width Δ_j, and center energy, $E_{0,j}$, such that $\rho(\omega) = \sum_j \frac{A_j \Delta_j}{(\hbar\omega - E_{0,j})^2 + \Delta_j^2}$, $j = 1,2 \ldots 5$. The rise towards 3eV manifests the interband absorption of gold.

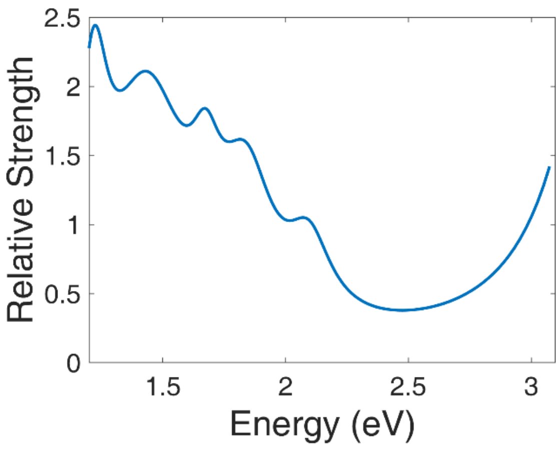

Figure S4: The plasmonic scattering spectrum, $\rho(\omega)$, used to fit all spectra of Fig. 1d.

b. Dependence on current - $\alpha(I)$

The values of α are obtained from the fit to of the measured signal to $S(\omega) = \alpha(I)\rho(\omega)\left(e\tilde{V} - \hbar\omega\right)$. It can be seen that α increases monotonously with I, and the dependence can be well approximated as linear. During the relatively long time over which each spectrum was taken, up to 8 minutes, the current through the device decreased gradually, probably due to the process of amorphization, which changes the electrical nature of our junctions. We record the current at the beginning and end of each measurement and define the average of the two as the current to be used for

modeling. The error bars in Figure S5 reflect the range over which the current changes in each voltage.

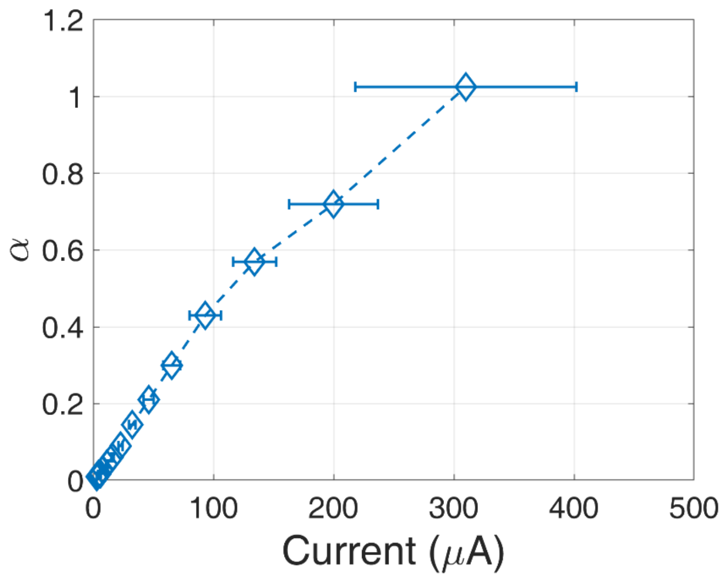

Figure S5: The fitted amplitude of the EDP signal, α, as a function of the measured current through the device, I, in the -1.7V to -3V range.

c. Fitting at finite temperatures

In the inset of Fig. 1d, we show how the spectra should behave at a zero-temperature approximation and at a finite temperature. The expression for $S(\omega)$ changes when temperature is included:

$$S(\omega,T) = \alpha\rho(\omega)\int_{\hbar\omega}^{\infty} d\omega' f_{FD}(\omega',T,\tilde{V}),$$

where $f_{\mathrm{FD}}(\omega) = \frac{1}{1+e^{\left(\frac{\hbar\omega - e\tilde{V}}{k_B T}\right)}}$ is the Fermi-Dirac distribution of the electrons in the gold layer,k_B is the Boltzmann constant, T is the electron temperature, and $\tilde{V}$ is the voltage drop across the junction.

3. Spectra and Images after Breakdown

a. Expanded data for the device described in the paper

The $2e^-$ tunneling emission is clearly observed in all of the spectra, exhibited as flat emission all the way to the end of our high-energy detection limit at 3.1 eV. It is worth noting that the $2e^-$ signal continues to rise with increasing voltage (and consequently – increasing current). We measure an intense signal of ~100 counts/s at 3.1eV at an applied bias of -8V. It is evident from the figure that the ratio of $2e^-$ tunneling to $1e^-$ increases dramatically.

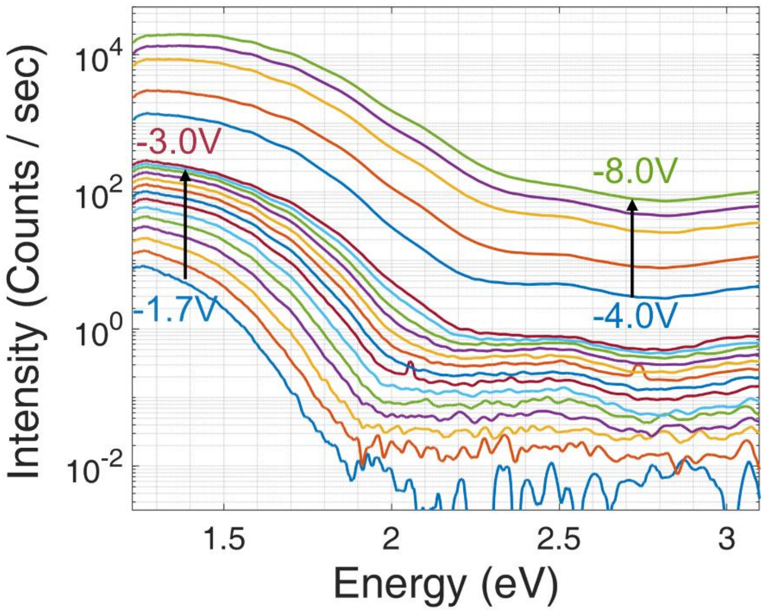

Figure S6: The emission spectrum of the device discussed in the paper for the voltage range -1.7V to -8V after breakdown has occurred. The intensity is presented on a log scale. The -1.7V to -3V range is separated by 100 mV, and the -4V to -8V range is separated by 1V.

b. Hot spot prevalence

The appearance of hot spots in our devices signals a permanent physical change. Following the set of images in Fig. S7 from -4.25V to -20V, it can be seen that each image is composed of the spots from the previous image as well as several new ones, all of which persist in the same location. This is true whether a higher or lower voltage is applied and can be seen from the final image in the succession: the spots that appear in the image of the device under -20V appear again when the voltage is lowered to -4V, even though they did not appear there when this voltage was originally applied. We find that this is true even if we turn the device off for several days – the number of spots and their locations are permanent.

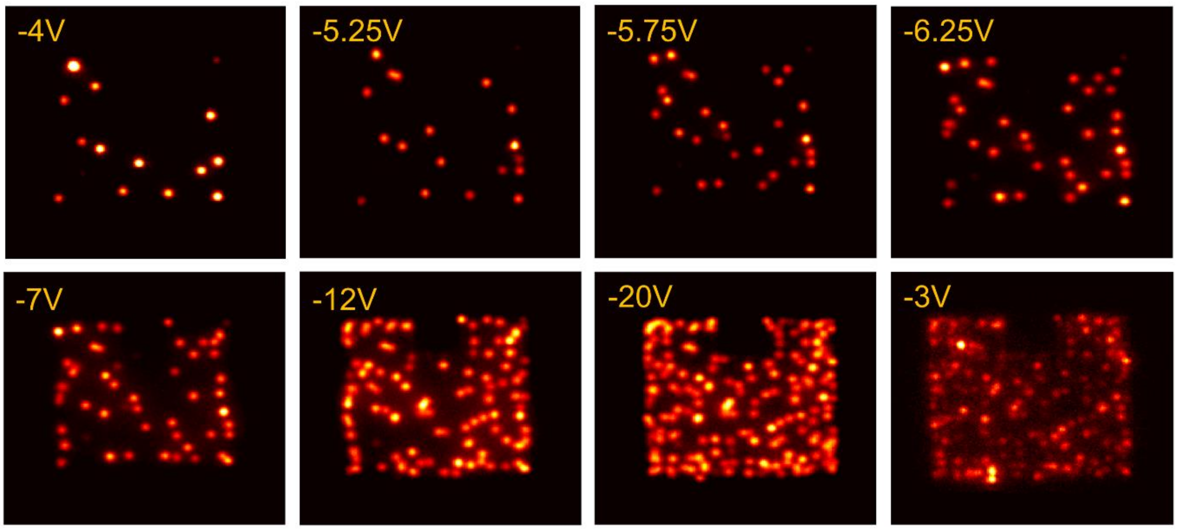

Figure S7: Formation of hot spots as the applied voltage on the device is increased.

c. Measurements of other devices after breakdown

In Fig. S8 we present the spectral and spatial emission from two devices ***after*** breakdown, and show that they depict the same behavior reported for the main device of the paper. We can see that the emission consists of diffraction-limited hot spots with intense emission. Additionally, as in the post-breakdown measurements presented in the main part of the paper, the spectra exhibit a peak at $\hbar\omega \approx 1.2 - 1.3\,eV$, corresponding to the a-Si band gap. The flattening of the spectra at $\hbar\omega > 1.3\,eV$, which manifests the accumulation of holes near the junction, is especially pronounced in Fig. 8a. Finally, one can observe the slow decay of the emission towards high energies, due to high electron temperature.

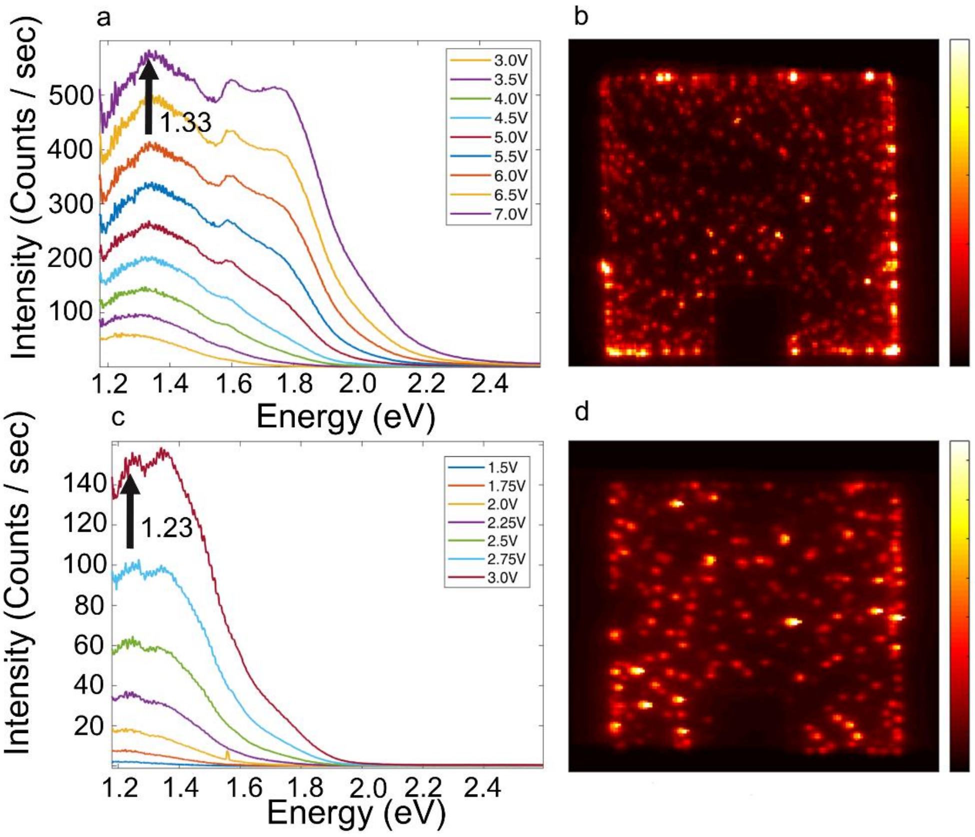

Figure S8: Spectra (a and c) and respective emission maps (b and d) of additional devices after breakdown. Arrows in (a) and (c) indicate the a-Si band gap.

__d. Band diagrams after breakdown (under hot spots)__

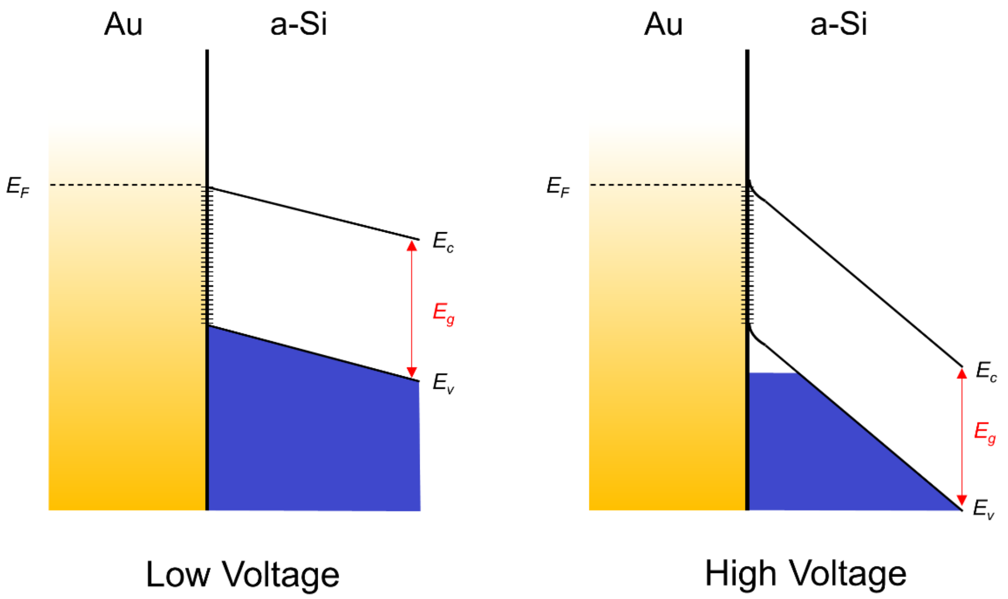

Figure S9: Local band diagram of the device directly under hot spots at high and low voltages.

Figure S9 depicts the band diagram at low and high voltage. As implied by the observed emission peak at 1.25eV (Fig. 4c), the conduction band is pinned to the gold Fermi level at the junction, and falls steadily towards its low value far away from the junction. Hence, the a-Si layer can be viewed as an insulating layer, on which the applied voltage drops. At high voltage, when the potential drop over the a-Si layer is a few Volts, a depletion layer of holes is formed near the junction. This depletion layer broadens the emission peak, and eventually develops at $V_{ext} < -4V$ into a flat region in the EDP spectrum (inset of Fig. 4c). It should be noted the maximal width of this plateau at the extreme condition of $V_{ext} = -20V$ is $\approx 200meV$, corresponding to an areal charge density $\sim 10^{13}cm^{-2}$.

4. Amorphization Measurements

a. Bright field STEM image of lamella at -20V

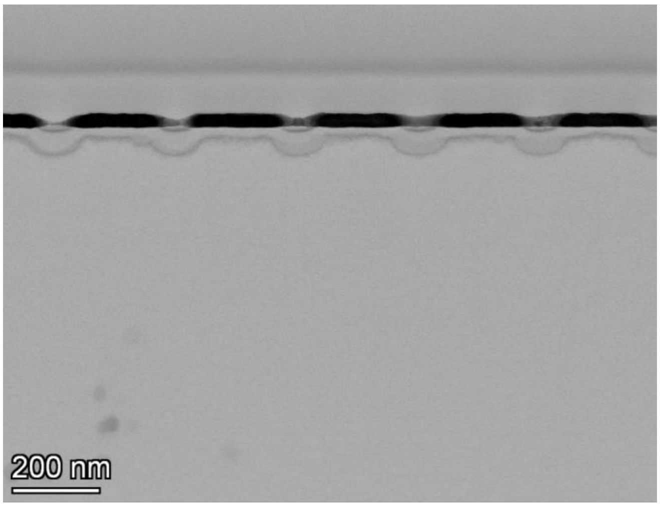

Figure S10: An expanded view of a STEM image of a cross-section of the device presented in Fig. 2b at -20V

In this “zoomed-out” presentation of the lamella of the device after a remarkably high voltage was applied to it, it is clear that the amorphization of the silicon follows the pattern of the Au electrode precisely. In this image of the cross-section, which is magnified at 65kx, there are six holes in the (black) Au electrode, under which six amorphization "wells" can be seen. It is evident that the effect persists throughout the entirety of the device and is not a localized anomaly. The deeper penetration of the amorphous layer into the silicon crystal in the regions not covered by gold indicates that these regions experience higher temperatures when current flows through the device. This implies that the heat radiation in these regions is less efficient than in the in the gold covered regions.

b. Amorphization depths

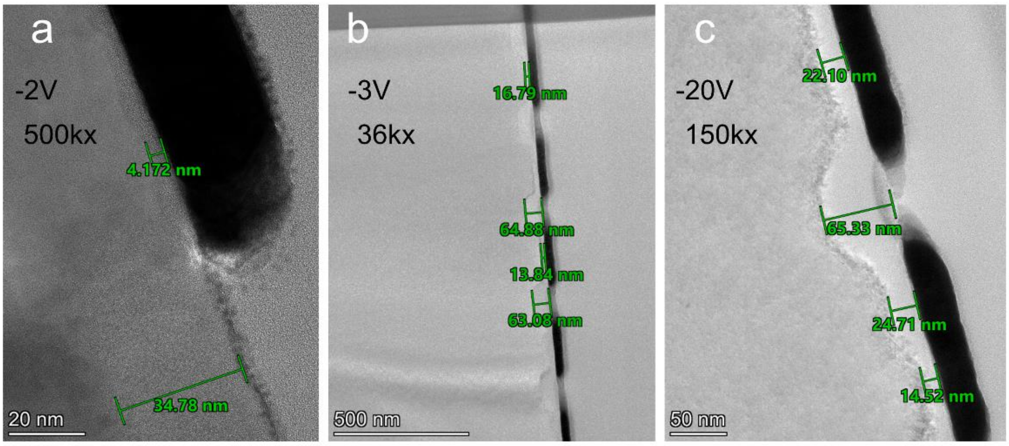

Figure S11: Angled BF TEM images of device cross-sections where -2V (a) -3V (b) and -20V (c) were applied.

We took measurements of the depth of a-Si along the lamellae under the holes and under the Au electrode. It can be seen that higher applied voltages, which are accompanied by larger currents flowing through the device and therefore higher temperatures, lead to a deeper penetration of the amorphization. At -3V, the amorphization depths are fairly identical to that of the -20V sample, and we conclude that the amorphization increases with voltage up to the transition voltage, after which it remains fairly constant. It should be noted that while the shape of the c-Si under the Au electrode appears to be flat, there are certain locations where there is a sudden deviation. We summarize our findings in the histograms below.

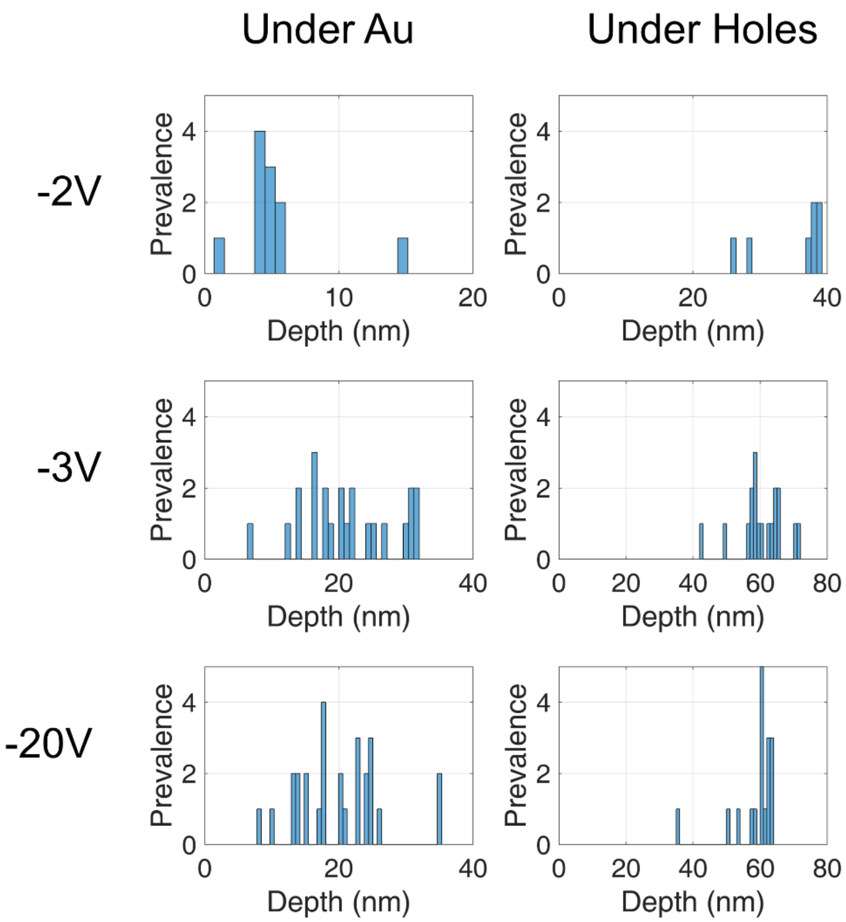

Figure S12: Histograms of the different amorphization depths for the -2V -3V, and -20V lamellae, separated into measurements recorded under the gold electrode (left) and under the holes (right).

A comparison of the depth distributions reveals that after applying -3V and -20V, they are centered around ~60 nm under the holes, and around ~20 nm under the gold, whereas the -2V device has a distribution that is centered around ~40 nm under the holes and only 5 nm under the gold electrodes.

c. Energy-dispersive X-ray spectroscopy

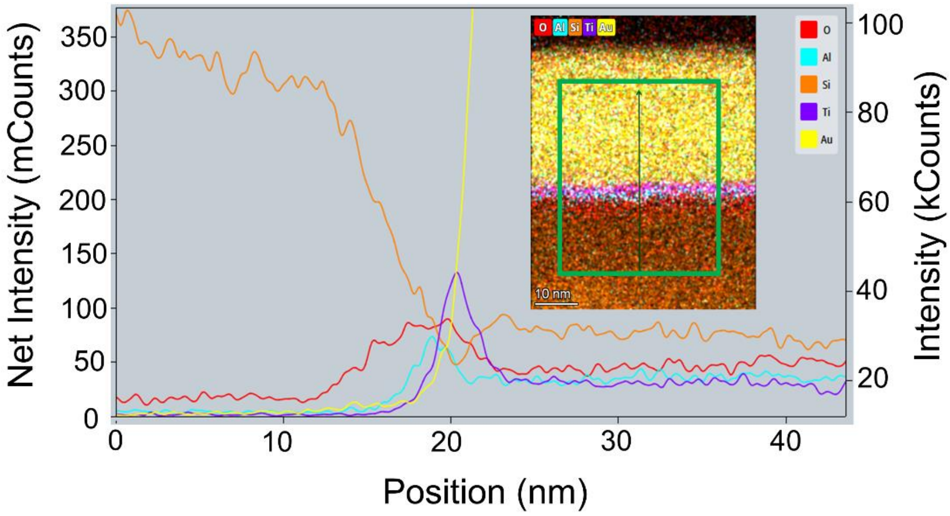

Figure S13: Elemental line profile along the tunnel junction. The inset shows the same image from Figure 2e, with the green box indicating the measurement area taken for the EDS profile and the arrow indicating the direction of the scan.

It can be seen from both the line profile and the elemental mapping that the tunnel junction remains intact and that that Au and Si regions remain separated with no diffusion of one into the other. We note that about 3.5 nm of the Si appear to be oxidized, but the EDS line profile of a control lamellae, which had no voltage applied to the device from which it was extracted, showed the same oxygen profile. We conclude that this is not due to a current or voltage-induced diffusion, and could very well be present in the silicon substrate before fabrication has begun.

The measurements were taken from a $\sim$110 nm thick lamellae, which lead to intense signals not only from the cross-section measured, but also the many layers of atoms behind the frontal layer. The slow decay of the silicon signal, for example, in the EDS scan (where one would expect an abrupt decay at the interface) is an effect of this limitation. Additionally, it would appear as if the Ti layer, which is only 1nm thick, is spread over a thicker range, $\sim 3-4$ nm, penetrating into the alumina region. This

spread reflects the limited resolution that can be obtained from a relatively thick lamellae. Nevertheless, it could be clearly seen that the peaks of the Ti distribution is displaced with respect to the Al peak, allowing us to assert the locations of each layer relative to each other: Si -> AlO_x -> Ti -> Au.

5. Miscellaneous

a. Spectra with and without noise filtering

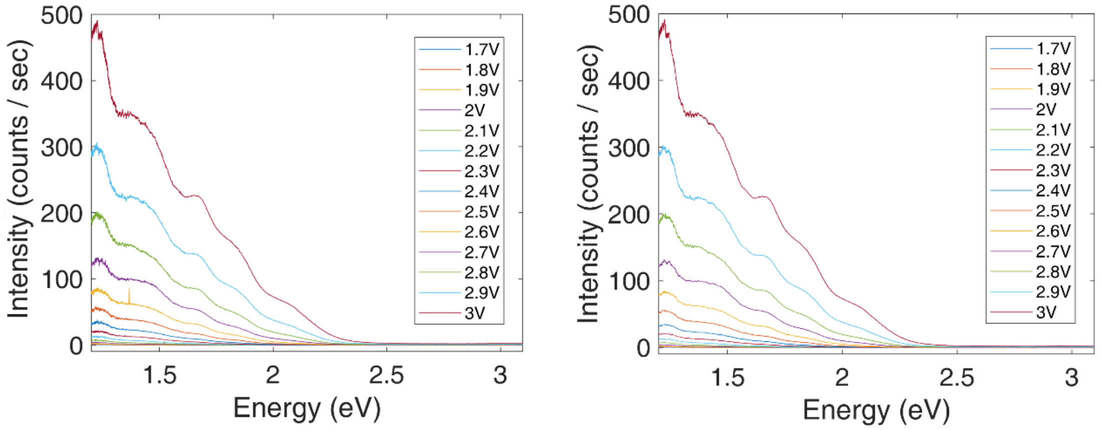

Figure S14: Spectra presented in 1d before (left) and after (right) filtering noise.

The spectra presented in the paper account for noise using a simple third-order 1D median filter. The data reflect a range of 700 nm and the averaging is done over ~ 5 nm.

b. AFM line scan of the alumina layer

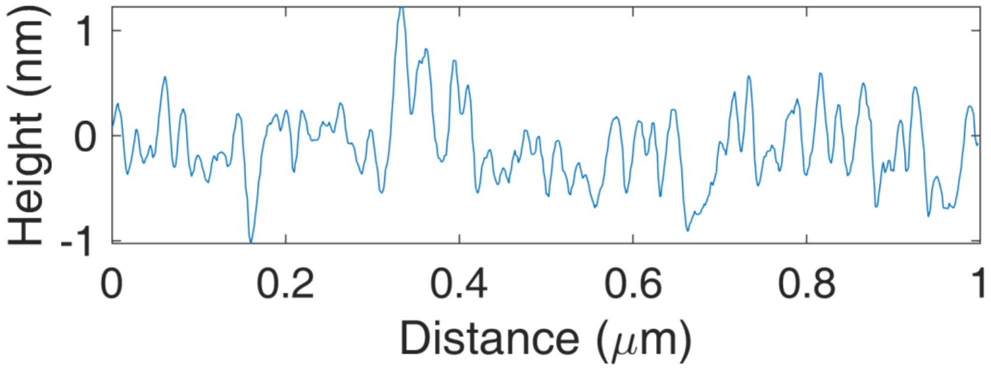

Figure S15: AFM scan of 1 μm of device after depositing 4 nm of AlO_x

We measure the uniformity of the alumina barrier after ALD deposition and find a standard deviation of roughly 3.6 Å. It can be seen that despite having an average standard deviation that is an order of magnitude smaller than the total deposited width, there are local minima of up to 1 nm. These localized defects are the first to break down when a high enough voltage is applied to the device.
